# Ovarian cancer ascites increase Mcl-1 expression in tumor cells through ERK1/2-Elk-1 signaling to attenuate TRAIL-induced apoptosis

**DOI:** 10.1186/1476-4598-11-84

**Published:** 2012-11-17

**Authors:** Nadzeya Goncharenko-Khaider, Isabelle Matte, Denis Lane, Claudine Rancourt, Alain Piché

**Affiliations:** 1 Département de Microbiologie et Infectiologie, Faculté de Médecine, Université de Sherbrooke, 3001, 12ième Avenue Nord, Sherbrooke, J1H 5N4, Canada

**Keywords:** Ovarian cancer, Resistance, Mcl-1, ERK1/2, TRAIL, Elk-1

## Abstract

**Background:**

Ascites may affect the progression of ovarian cancer (OC). In particular, soluble factors present in OC ascites can create a protective environment for tumor cells that promote *de novo* resistance to drug- and death receptor-induced apoptosis. However, the underlying molecular mechanisms responsible for ascites-induced drug resistance are not well characterized.

**Methods:**

Using human OC cell lines and tissues microarrays of human OC biopsies, we assessed the mechanism by which OC ascites increase Mcl-1 expression using Western blots, chemical inhibitors of ERK and small-inhibitory RNA treatments.

**Results:**

In the present study, we found that both Mcl-1 mRNA and protein levels were upregulated within 2 h upon treatment of OC cells with ascites obtained from women with advanced OC. In contrast, the expression of other Bcl-2 family antiapoptotic members such as Bcl-2 and Bcl-X_L_ was not affected by ascites. An increase of Mcl-1 expression was consistently observed across different ascites from women with advanced serous OC. The knockdown of Mcl-1 significantly blocked ascites-induced Mcl-1 upregulation and ascites-mediated inhibition of TRAIL-induced apoptosis. Ascites induced a rapid phosphorylation of ERK1/2 and Elk-1 transcription factor. Furthermore, we found that ERK1/2 inhibition or Elk-1 knockdown was sufficient to block ascites-induced Mcl-1 expression. In high grade serous OC, we found a positive correlation between phosphorylated ERK1/2 and Mcl-1 expression.

**Conclusions:**

These results indicate that ascites-induced ERK1/2/Elk-1 signaling is critical for Mcl-1 expression and for the ascites-mediated attenuation of TRAIL-induced apoptosis. The ERK1/2/Elk-1/Mcl-1 pathway represents a novel mechanism by which ascites induce *de novo* TRAIL resistance in OC cells.

## Background

Ovarian cancer (OC) is the fifth cause of cancer-related death in women, the second most common gynecological cancer, and the leading cause of death from gynecological malignancies [1-3]. High grade serous OC is the most common subtype of OC and over 70% of these patients present with late stage diseases and dissemination of tumor implants throughout the peritoneal cavity [4,5]. Despite initial aggressive treatment, the five-year survival of patients with late stage disease remains at < 30%, a figure that has not changed for the past 30 years [2]. This is related, at least in part, to the persistence of minimal residual disease after chemotherapy, which contributes to shorter progression-free survival [6,7]. The tumor environment is being increasingly recognized as an important contributor of tumor progression [8-10] as it may facilitate the survival [11-14], differentiation and proliferation of tumor cells [15,16]. Furthermore, ascites create a protective environment for ovarian tumor cells that inhibit drug-induced apoptosis (*de novo* resistance) [13,17]. Ascites are heterogenous fluids that display marked differences in their levels of soluble factors but some of these factors can potentially activate an array of signaling pathways [18-24]. The demonstration that ascites with prosurvival properties are associated with a shorter progression-free survival in patient with OC underscores the critical role of ascites in OC progression [6]. The molecular changes in tumor cells induced by ascites that result in resistance have not been well characterized. It is important to define the contribution of each pathway both to fully understand cell survival signaling and to validate individual pathways as therapeutic targets.

Activation of the Raf/MEK/ERK pathway has been often associated with the promotion of cell proliferation but also represents, in addition to the PI3K/Akt pathway, an important survival signaling pathway in many tumor cells [25]. The Raf/MEK/ERK pathway promotes survival through the inhibition of the apoptotic cascade by controlling the expression or the activity of Bcl-2 family members [26,27]. There is evidence that the ERK pathway activation increases the expression of prosurvival Bcl-2 proteins, notably Mcl-1, by promoting *de novo* gene expression [26,28-30]. The relative expression of Mcl-1 in tumor cells can be regulated at the transcriptional level or through post translational modifications by ERK [31]. In addition to the ERK signaling, the PI3K/Akt pathway has been found to be critical for Mcl-1 expression [32-34]. The importance of Mcl-1 in mediating tumor necrosis factor-related apoptosis-inducing ligand (TRAIL) resistance has been well documented in different cell types [35]. Overexpression of Mcl-1 can attenuate apoptosis induced by TRAIL [36]. Conversely, downregulation of Mcl-1 by siRNA enhances TRAIL-mediated cell death [37].

TRAIL belongs to the TNF family of cytokines and has emerged as a promising anticancer agent, because of its ability to selectively induce apoptosis in a broad host of tumor cells [35,38]. TRAIL binding to its receptors (TRAIL-R1 and TRAIL-R2) initiates the extrinsic pathway, resulting in recruitment of the adapter protein Fas-associated death domain (FADD) and procaspase-8 in the death inducing signaling complex (DISC). In some cells (type I cells), the apoptotic signal from active caspase-8 is sufficient to activate downstream effector caspases and induce apoptosis [39]. However, in other cell types, such as OC cells, the apoptotic signal must be further amplified by engaging the intrinsic (mitochondrial) pathway [39]. In this context, caspase-8 cleaves Bid to generate an active tBid, which in turn activates proapoptotic Bax or Bak proteins, and induces mitochondrial outer membrane permeabilization (MOMP). The mitochondria then releases proapoptotic factors that promote effector caspase activation. Overexpression of antiapoptotic Bcl-2 family members, including Bcl-2, Bcl-XL and Mcl-1 is associated with TRAIL resistance in type II cells, because of their ability to prevent tBid-induced MOMP [40].

In this study, we demonstrate that transcriptional upregulation of Mcl-1 by OC ascites is mediated by an ERK-dependent activation of the transcription factor Elk-1. Moreover, we demonstrate that upregulation of Mcl-1 has a significant role in ascites-mediated attenuation of TRAIL-induced apoptosis.

## Results

### OC ascites upregulate Mcl-1 expression

**Figure 1:**
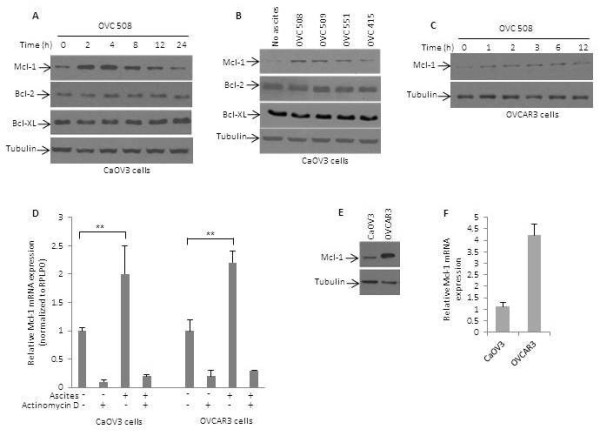
**OC ascites stimulate Mcl-1 protein and mRNA levels.** (**A**) Time course of Mcl-1 protein expression in CaOV3 cells following addition of 10% OVC508 ascites to the cell culture media. Cells were incubated with complete media for 24 h before the addition of ascites. Immunoblots were probed with anti-Mcl-1, anti-Bcl-2, anti-Bcl-X_L_ and anti-tubulin (as a loading control). (**B**) Immunoblot analysis of Mcl-1, Bcl-2, Bcl-X_L_ and tubulin expression in CaOV3 cells incubated with different OC ascites (10%). Lysates were obtained 2 h following the addition of ascites. (**C**) Time course of Mcl-1 expression in OVCAR3 cells following addition of 10% OVC508 ascites. (**D**) Real-time PCR analysis of Mcl-1 transcript levels in the presence or absence of OVC508 ascites (10%) and actinomycin D. Results were standardized using primers of the housekeeping gene RPLPO. Results are expressed as fold change relative to basal levels observed in cells incubated in the absence of ascites. (**E**) Immunoblot analysis of basal levels of Mcl-1 protein in CaOV3 and OVCAR3 cells. (**F**) Quantitative real-time PCR of Mcl-1 mRNA expression levels. Expression data were normalized to internal RPLPO RNA expression. Data represents the mean of two experiments ± SEM. ** indicates *P* < 0.001.

Previous studies have shown that OC ascites obtained from women with advanced disease attenuate TRAIL-induced apoptosis [13,17], and ascites with prosurvival activity negatively affect progression-free survival [6]. One of the mechanisms by which ascites attenuate TRAIL-induced apoptosis in OC cells is through engagement of αvβ5 integrin and subsequent activation of Akt survival signaling pathway which results in the upregulation of caspase-8 inhibitor c-FLIPs [13,17]. However, given the relative abundance of survival factors in ascites, other signaling pathways likely contribute to promote TRAIL resistance. Microarray data analysis of OC cells (CaOV3) exposed to ascites revealed that Mcl-1 was one of the genes differentially upregulated (data not shown). Because several studies in various cancer types have demonstrated that overexpression of the antiapoptotic protein Mcl-1 may promote TRAIL resistance [35], we examined the contribution of Mcl-1 to ascites-induced TRAIL resistance in the TRAIL-sensitive OC cell line CaOV3 and OVCAR3. OVCAR3 is an ovarian carcinoma cell line isolated from malignant ascites that is resistant to clinically relevant concentrations of cisplatin but remains sensitive to TRAIL-induced apoptosis. CaOV3 is also an ovarian carcinoma cell line isolated from a patient with advanced disease. Both cell lines have been extensively used by our group and the TRAIL signaling cascade has been well characterized [41-43]. In addition, we have previously shown that TRAIL-induced apoptosis is inhibited by OC ascites in these cell lines [13,17]. We first examined Mcl-1 protein and mRNA levels in CaOV3 and OVCAR3 cell lines following treatment with ascites. As shown in Figure 1A, CaOV3 cells demonstrated a marked increase of Mcl-1 protein within 2 h of exposure to OVC508 ascites, which remained elevated for up to 12 h. Expression of antiapoptotic proteins Bcl-2 and Bcl-X_L_ remained however unchanged following treatment with OVC508 ascites. To ensure that ascites effect on Mcl-1 was not limited to a single ascites, additional ascites were tested and all consistently upregulated Mcl-1 at 2 h, albeit to different degrees, without affecting Bcl-2 or Bcl-X_L_ (Figure 1B). Mcl-1 protein was also upregulated by ascites in the OVCAR3 cell line (Figure 1C and Additional file 1: Figure S1A). To determine whether Mcl-1 expression changes were the result of increased transcription or altered protein stability, we examined Mcl-1 mRNA levels in CaOV3 and OVCAR3 cells at 2 h following exposure to ascites. Mcl-1 mRNA levels, as determined by quantitative real time PCR, were upregulated by at least two fold in both CaOV3 and OVCAR3 cells, which could be inhibited by pretreatment with actinomycin D (Figure 1D and Additional file 1: Figure S1B) indicating that this was due to transcriptional increase, rather than a change in the mRNA stability. This was further supported by the observation that ascites did not alter Mcl-1 protein stability (Additional file 1: Figure S1C). Indeed, when levels of Mcl-1 were depleted in OVCAR3 cells incubated for 4 h in the presence of cycloheximide (10 μg/ml) to block *de novo* protein biosynthesis, the turnover of Mcl-1 was not affected by the addition of ascites. Of note, the magnitude of Mcl-1 upregulation was not as strong in OVCAR3 cells when compared to CaOV3 cells but OVCAR3 cells expressed higher basal levels of Mcl-1 protein (Figure 1E) and mRNA (Figure 1F). All together, these data demonstrate that OC ascites upregulate Mcl-1 expression in OC cells.

### Mcl-1 contributes to ascites-induced attenuation of TRAIL-mediated apoptosis

**Figure 2:**
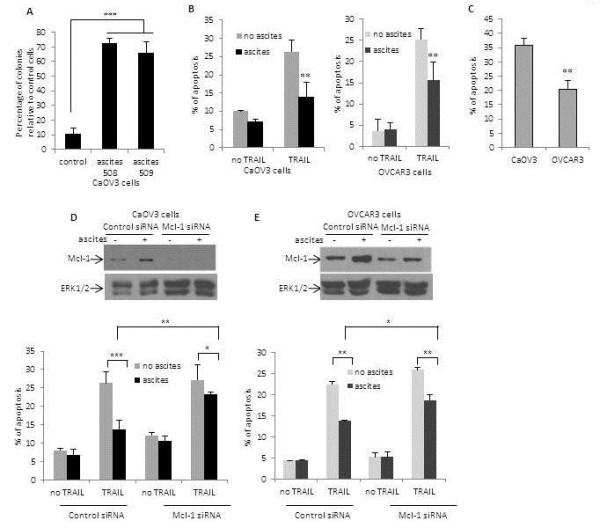
**Mcl-1 inhibition abrogates the prosurvival activity of ascites.** (**A**) The effect of ascites on CaOV3 cell survival was assessed by long term assays where cells were exposed to TRAIL (50 ng/ml) for 24 h in the absence (control) or presence of ascites (10%). After 14 days colonies were counted and expressed as percentage of colonies obtained in the absence of TRAIL. (**B**) The percentage of apoptosis was measured as percentage of hypodiploid cells assessed by flow cytometry analysis during exposure to TRAIL (50 ng/ml for CaOV3; 100 ng/ml for OVCAR3) for 24 h in the presence or absence of OVC508 ascites. (**C**) CaOV3 and OVCAR3 cells were treated with a similar concentration of TRAIL (50 ng/ml) and apoptosis was assessed by flow cytometry 24 h later. (**D**) CaOV3 and (**E**) OVCAR3 cells were transfected with Mcl-1 or control siRNA in the presence or absence of OVC508 ascites. Mcl-1 knockdown was confirmed by immunoblot analysis 24 h after transfection and ERK1/2 was used as a loading control. Apoptosis was assessed 24 h following siRNA transfection in the presence or absence of TRAIL (50 ng/ml). Data shown are means ± SEM derived from three independent experiments. * indicates *P* < 0.01, ** *P* < 0.001, *** *P* < 0.0001.

Given its antiapoptotic activity, Mcl-1 could contribute to ascites-induced attenuation of TRAIL-induced apoptosis. Thus, we investigated whether Mcl-1 inhibition can alter the prosurvival activity of OC ascites. First, CaOV3 cells were incubated with ascites in the presence or absence of TRAIL (50 ng/ml) for 24 h. Long term cell survival was assessed by determining the fraction of surviving colonies after two weeks. As shown in Figure 2A, the addition of OVC508 or OVC509 ascites to CaOV3 cells significantly (*P* < 0.0001) enhanced the fraction of survival cells. When apoptosis was determined by measuring the sub-G1 DNA content for CaOV3 and OVCAR3 cells incubated with ascites, we observed a 38% to 48% decreased of TRAIL-induced apoptosis (*P* < 0.001) confirming that ascites attenuate TRAIL-mediated cytotoxicity (Figure 2B). These data confirmed that pretreatment with ascites attenuates TRAIL-induced apoptosis in OC cells.

When CaOV3 and OVCAR3 cells were compared directly, the level of TRAIL-induced apoptosis was higher in CaOV3 cells (Figure 2C), consistent with the observation that CaOV3 cells expressed lower basal level of Mcl-1 (Figure 1E and 1F). To further assess the role of Mcl-1 in TRAIL resistance, CaOV3 cells were transfected with Mcl-1 or control siRNA and expression of Mcl-1 was assessed by immunoblot at 24 h and 48 h post transfection. Mcl-1 protein was efficiently downregulated by Mcl-1 siRNA in CaOV3 cells (Additional file 1: Figure S2A). Importantly, transfection of CaOV3 and OVCAR3 cells with Mcl-1 siRNA completely abrogated ascites-induced Mcl-1 upregulation in both CaOV3 (Figure 2D) and OVCAR3 cells (Figure 2E). Of note, the expression of antiapoptotic protein Bcl-2 and Bcl-X_L_ remained unaffected by Mcl-1 siRNA (Additional file 1: Figure S2B). Mcl-1 depletion significantly blocked the prosurvival activity of ascites in CaOV3 and OVCAR3 cells. As shown in Figure 2D, TRAIL (50 ng/ml) induced apoptosis in CaOV3 cells whereas the presence of ascites, as expected, significantly inhibited TRAIL-induced apoptosis (*P* < 0.001). In CaOV3 cells transfected with Mcl-1 siRNA, the protective effect of ascites was almost completely abrogated. The transfection of Mcl-1 siRNA in OVCAR3 cells also significantly (*P* < 0.01) inhibited the protective effect of ascites albeit to a lesser extend (Figure 2D). This could be related to the observation that the Mcl-1 siRNA did not completely block Mcl-1 expression in OVCAR3 cells.

### OC ascites upregulate Mcl-1 through ERK1/2 signaling

**Figure 3:**
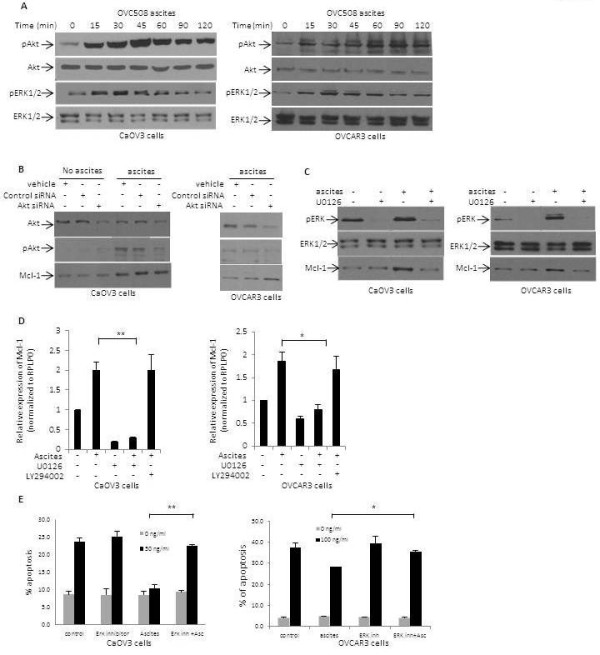
**ERK1/2 signaling regulates the Mcl-1 expression.** (**A**) Immunoblot analysis of Akt, phospho-Akt, ERK1/2 and phospho-ERK1/2 in CaOV3 and OVCAR3 cells following the addition of 10% OVC508 ascites. (**B**) Expression levels of Akt, phospho-Akt and Mcl-1 in vehicle, Akt or control siRNA-transfected CaOV3 and OVCAR3 cells 24 h post-transfection in the presence or absence of OVC508 ascites. (**C**) Immunoblot analysis of ERK1/2, phospho-ERK1/2 and Mcl-1 in OVC508 ascites- and U0126-treated OVCAR3 and CaOV3 cells 24 h post treatement. (**D**) Real-time PCR analysis of Mcl-1 transcript levels in CaOV3 and OVCAR3 cells. Cells were treated with either ascites (OVC508), MEK1/2 inhibitor U0126 or Akt inhibitor LY294002 and RNA was extracted 4 h later. Results were standardized using primers of the housekeeping gene RPLPO. Results are expressed as fold change relative to basal levels observed in cells incubated in the absence of ascites. (**E**) Apoptosis was assessed 24 h following incubation with U0126 in the presence or absence of TRAIL 50 ng/ml for CaOV3 cells and 100 ng/ml for OVCAR3 cells. Data shown are means ± SEM derived from three independent experiments. * indicates *P* < 0.001, ** *P* < 0.0001.

Activation of both ERK1/2 and Akt pathways has been linked to the transcriptional regulation of Mcl-1 [26,28-30,32,34]. Previous studies demonstrating Akt activation by ascites [13,17] prompted us to investigate whether Akt and ERK1/2 were involved in ascites-mediated upregulation of Mcl-1 expression. First, we examined the phosphorylation of Akt and ERK1/2 overtime and found that both Akt and ERK1/2 were activated by ascites (Figure 3A and Additional file 1: Figure S3). siRNA-mediated inhibition of Akt in both CaOV3 and OVCAR3 cells however did not altered ascites-mediated up-regulation of Mcl-1 expression (Figure 3B). The chemical inhibitor of Akt LY294002 produced similar results (data not shown) suggesting that ascites-mediated Mcl-1 up-regulation is not dependent of Akt activation. In contrast, when ERK1/2 activation was inhibited by the specific MEK1/2 inhibitor U0126 [44], ascites-mediated upregulation of Mcl-1 protein was substantially blocked in both CaOV3 and OVCAR3 cells (Figure 3C). Consistent with these results, U0126 significantly blocked the transcriptional upregulation of Mcl-1 by ascites in CaOV3 and OVCAR3 cells (Figure 3D). In contrast, the inhibition of Akt by LY294002 had no impact on ascites-mediated transcriptional upregulation of Mcl-1 in OC cells (Figure 3D). Because Mcl-1 contributes to ascites-mediated protection from TRAIL-induced apoptosis, we examined whether ERK1/2 has a similar role. As expected, ERK1/2 inhibition by U0126 significantly blocked ascites-mediated protection from TRAIL-induced apoptosis (Figure 3E). These data demonstrate that ERK1/2 activation upregulates Mcl-1 expression and contributes to ascites-mediated attenuation of TRAIL-induced apoptosis.

### Ascites activates Elk-1 transcription factor via ERK1/2 pathway

**Figure 4:**
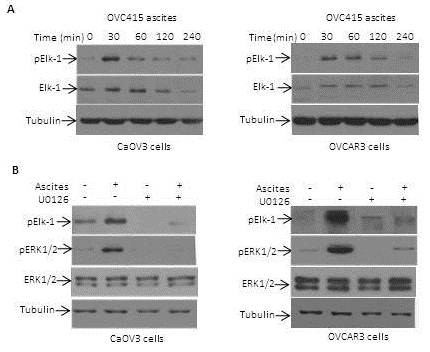
**Elk-1 is activated by ascites and its activation is controlled by ERK1/2.** (**A**) Cells were treated with OVC415 ascites (10%) for up to 240 min and the expression and phosphorylation of Elk-1 was assessed by immunoblot. Activation of Elk-1 was detected with an anti-phospho-Elk-1 antibody specific for Ser383. Tubulin was used as a control for loading. (**B**) Cells were treated with U0126 for 1 h before the addition of OVC415 ascites (10%). Lysates were obtained 1 h after the addition of ascites and immunoblot analysis was performed with anti-phospho-ERK1/2 and anti-phospho-Elk-1 antibodies. Representative data from three independent experiments.

Previous studies have shown that ERK1/2 can directly phosphorylate and activate many transcription factors including Elk-1 in breast cancer cells [28]. ERK1/2 activation promotes Elk-1 phosphorylation at Ser383 and its activation. We therefore determine whether ascites treatment of OC cells resulted in activation of Elk-1. As shown in Figure 4A, the treatment of CaOV3 and OVCAR3 cells with OVC415 ascites resulted in Elk-1 phosphorylation (Ser383) within 30 min and phosphorylation declined thereafter. This was similar to the kinetic of ERK1/2 that was observed in CaOV3 and OVCAR3 cells (Figure 3A). To ensure that ascites-induced Elk-1 phosphorylation was not limited to a single ascites, CaOV3 and OVCAR3 cells were treated with OVC508 and Elk-1 activation was assessed. As shown in Figure 4B, treatment with OVC508 also resulted in Elk-1 activation. Pretreatment with U0126 prevented both ascites-induced ERK1/2 and Elk-1 phosphorylation in CaOV3 and OVCAR3 cells (Figure 4B). These data demonstrate that ascites-induced Elk-1 activation is ERK1/2-dependent in OC cells.

### Ascites-dependent Elk-1 activation is responsible for Mcl-1 regulation

**Figure 5:**
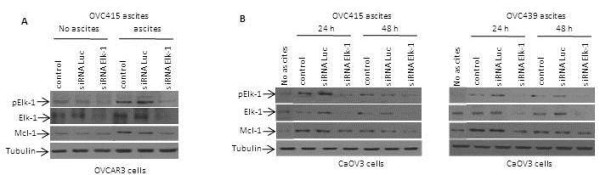
**Elk-1 activation is essential for the control of Mcl-1 expression.** (**A**) OVCAR3 cells were transfected with vehicule (control), control siRNA luciferase (siRNA Luc) or Elk-1 specific siRNA (siRNA Elk-1) and 24 h later cells were treated with OVC415 ascites for 1 h or left in medium with FBS. Lysates were obtained 1 h after the stimulation with ascites and immunoblots were probed with anti-phospho-Elk-1, anti-Elk-1 and anti-Mcl-1 antibodies. Tubulin was used as a loading control. (**B**) CaOV3 cells were transfected with vehicule, control siRNA Luc or Elk-1 specific siRNA and 24 or 48 h later cells were stimulated with either OVC415 (left panel) or OVC439 ascites (right panel). Lysates were obtained and immunoblotted with anti-Elk-1, anti-Mcl-1, anti-phospho-Elk-1 and anti-tubulin. Representative data from three independent experiments.

To determine whether ascites-induced activation of Elk-1 transcription factor is responsible for Mcl-1 upregulation, OVCAR3 cells were transfected with Elk-1 or control siRNA and the expression of Elk-1 and Mcl-1 were determined 24 h later by immunoblot. As shown in Figure 5A, the knockdown of Elk-1 inhibited upregulation of Mcl-1 by ascites indicating a critical role of Elk-1 in Mcl-1 upregulation. Similar to what we observed in OVCAR3 cells, CaOV3 cells transfected with Elk-1 siRNA displayed reduced Mcl-1 expression at 24 h and 48 h following treatment with OVC415 and OVC439 ascites (Figure 5B).

### Ascites-mediated ERK/Ekl-1 signaling is independent of FAK activation

**Figure 6:**
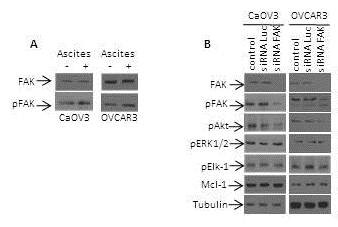
**Ascites-induced ERK1/2 activation is independent from the activation of FAK.** (**A**) Immunoblot analysis of expression and activation of FAK in both CaOV3 and OVCAR3 cells in the presence of ascites (OVC415). (**B**) Cells were transfected with vehicle (control), control luciferase siRNA (siRNA Luc) or FAK siRNA (siRNA FAK) and 24 h later cells were stimulated with OVC415 ascites for 1 h. Lysates were then obtained and analyzed by immunoblot for total and phospho-FAK (Tyr397), phospho-Akt (Ser473), phospho-ERK1/2, phospho-Elk-1 and Mcl-1. Representative data from three independent experiments.

It has been previously shown that OC ascites induce a α6β1 integrin-dependent activation of ERK1/2 pathway [24] and a αvβ5 integrin-mediated activation of Akt pathway [13]. The engagement of integrins to the extracellular matrix (ECM) components triggers a signaling cascade that leads to the activation of focal adhesion kinase (FAK), one of the earliest events that immediately follows integrin-ECM component engagement. In this context, we previously showed that ascites induce a rapid FAK activation [13]. Thus, we assessed whether FAK was involved in ascites-mediated activation of ERK1/2/Elk-1 signaling. To this end, CaOV3 and OVCAR3 cells were transfected with FAK or control siRNA and cells were treated with ascites. Figure 6 shows that siRNA-mediated FAK knockdown inhibited ascites-induced Akt activation as we have previously reported [13]. In contrast, ERK1/2 activation was not affected by FAK knockdown. Consistent with this observation, Elk-1 activation and Mcl-1 expression remained unaffected by FAK knockdown. These data suggest that integrin/FAK signaling is not critical for Mcl-1 upregulation.

### Activated ERK1/2 correlates with Mcl-1 expression in high grade serous OC (HGSOC)

**Figure 7:**
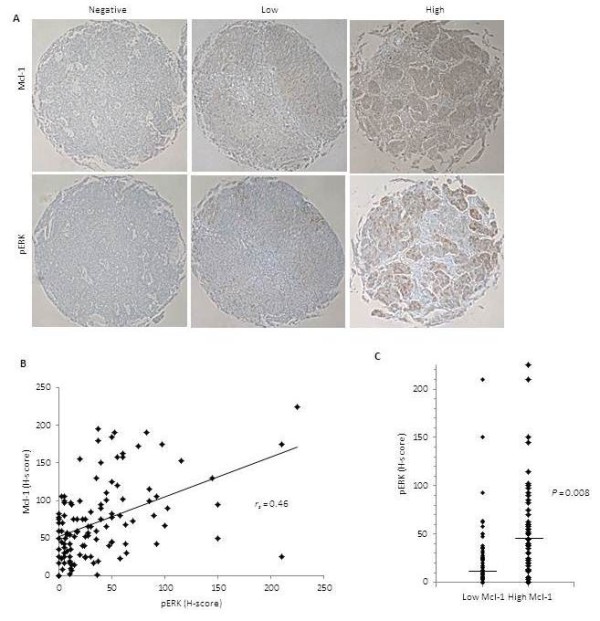
**Activated ERK1/2 correlates with Mcl-1 expression in HGSOC samples.** A TMA containing samples from 120 HGSOC was stained by immunohistochemistry with antibodies specific for phospho-ERK1/2 and Mcl-1. Tumors were scored using the H-score method. (**A**) Representative images of immunoperoxidase-stained tissue for phospho-ERK1/2 and Mcl-1 demonstrating either negative staining (0), low intensity (1) or high intensity staining (3). (**B**) Correlation chart for Mcl-1 and ERK between their H-scores as determined by immunohistochemistry. (**C**) H-score for phospho-ERK1/2 separated into two groups based on the median Mcl-1 H-score of 62.5.

To determine whether our *in vitro* findings were clinically relevant in human ovarian tumors, we assessed if the ERK1/2-dependent regulation of Mcl-1 expression in CaOV3 and OVCAR3 cell lines correlated in HGSOC, the most common subtype of OC. We initially performed a pilot study on 20 HGSOC samples to determine the optimal conditions for antibody staining against Mcl-1, phospho-ERK1/2 and phospho-Elk-1. Despite several attempts, we were unable to obtained consistent staining for phospho-Elk-1. Based upon this pilot study, we examined the relationship between phospho-ERK1/2 and Mcl-1 expression in a tissue microarray (TMA) of HGSOC provided by the Pan-canadian platform for the development of biomarker-driven subtype specific management of ovarian carcinoma (COEUR study). The TMA consisted of 120 HGSOC samples and each tumor was represented by two separate spots on the TMA. The immunostaining was scored using a 0–3 scoring system (H-score). Representative images from the sampled tumors demonstrate that regions within individual section expressing Mcl-1 also have positive phospho-ERK1/2 staining (Figure 7A). The data was analyzed by plotting the scores as an XY scatter and performing a Spearman correlation test (Figure 7B). We found a statistically significant positive correlation between the phosphorylation of ERK1/2 and Mcl-1 expression (*r*_*s*_ = 0.46, *P* = 0.015). The tumors were separated into two groups based on the median Mcl-1 H-score of 62.5. Samples with a score < 62.5 were classified as low Mcl-1 and those with a score > 62.5 were classified as high Mcl-1 (Figure 7C). The median phospho-ERK1/2 for the low Mcl-1 group was 12 and the median for the high Mcl-1 group was 46, a difference that was statistically significant using a Mann–Whitney test (*P* = 0.008). These data support the regulation of Mcl-1 expression by the ERK1/2 pathway in HGSOC.

## Discussion

The development of resistance to chemotherapy remains a major problem with OC. Indeed, the poor prognosis is usually attributed to the occurrence of resistance. Defects in the apoptotic cascade have been commonly associated with resistance in OC cells. Although a number of mechanisms have been proposed for OC cells, most studies were performed in unicellular models and did not take into account the interactions that exist between the host and tumor cells. Unlike most other solid cancers where the stroma surrounding tumor cells constitutes the tumor environment, ascites that develop during OC progression represent a unique form of tumor environment. Indeed, soluble factors in ascites create a proinflammatory environment that promotes *de novo* resistance [13,17]. Available evidence suggests that soluble factors in the tumor environment engage cell surface receptors to activate survival pathways [13]. This study extends our previous findings that ascites-induced activation of the Akt pathway attenuates TRAIL-induced apoptosis [13,17] by showing that ERK1/2/Elk-1 signaling is responsible for the transcriptional increase of Mcl-1, which in turn contributes to ascites-mediated inhibition of TRAIL-induced apoptosis in OC cells. Our results show that ascites induce a rapid activation of Akt and ERK1/2 but only that ERK1/2 activation is associated with Mcl-1 upregulation in tumor cells. Moreover, our results demonstrate that Mcl-1 upregulation is one of the mechanisms by which ascites protect OC cells from against TRAIL-induced apoptosis.

Although we have previously reported that one malignant ascites (COV2) induced the phosphorylation of Akt but not ERK [17], further works, as shown here and by other groups [24], have demonstrated that ERK activation by various OC ascites is a common findings. Similar observations have been made for the activation of the Akt pathway by ascites. Many ascites have the ability to activate this pathway [6,13,17] but it appears that some OC ascites are unabled to increase Akt phosphorylation in OC cell lines [6,13]. This is believed to be related to the heterogeneity of OC ascites.

TRAIL cytotoxicity in OC cells relies on the activation of both the extrinsic and the intrinsic apoptotic pathways [39]. These two pathways are interconnected, and in OC cells, the proapoptotic Bcl-2 family member Bid is a critical regulator of TRAIL resistance that connects both pathways by promoting mitochondrial activation [41]. Antiapoptotic Bcl-2 family proteins, such as Bcl-2, Bcl-X_L_ and Mcl-1, have a critical role in regulating the balance between survival and death signals at the mitochondrial level. Although Bcl-X_L_ may promote the survival of OC cells [42,45], the importance of Mcl-1 in OC survival has not been well established. Higher expression of Mcl-1 in OC compared to adenomas or normal ovaries has been reported [46-48], and was, in some studies, associated with poor prognosis [47]. Our study shows that Mcl-1, but not Bcl-2 nor Bcl-X_L_, is upregulated by OC ascites. Mcl-1 is a downstream target of activated ERK signaling and is important for survival of OC cells in response to TRAIL since siRNA inhibition of Mcl-1 significantly attenuates ascites-mediated resistance to TRAIL.

Ascites-induced signaling events trigger activation of both the Akt and the ERK1/2 pathways. We have previously shown that ascites-mediated Akt activation attenuates TRAIL-induced apoptosis in CaOV3 cells [17]. Ascites activate Akt, which in turn up-regulate the expression of cFLIPs, a caspase-8 inhibitor. The treatment of CaOV3 cells with PI3K/Akt inhibitors partially blocks ascites-mediated survival [17]. Activation of the PI3K/Akt pathway thus represents one way by which ascites confer resistance to TRAIL-induced apoptosis. The present study suggests that ERK1/2 pathway mediates the transcriptional upregulation of Mcl-1. Unlike inhibition of ERK1/2, blocking Akt pathway did not alter ascites-induced upregulation of Mcl-1. This is evidenced by the lack of effect of Akt downregulation by siRNA and Akt inhibition by LY294002 on Mcl-1 expression. In contrast, U0126-mediated inhibition of ERK1/2 readily decreased Mcl-1 at the transcriptional level, and promoted TRAIL-induced apoptosis in OC cells. These results indicate that ERK1/2, but not Akt pathway, plays a determining role in ascites-induced Mcl-1 expression. The ERK1/2 pathway has been previously reported to regulate Mcl-1 transcription in other cell types [26,28-30]. In addition, the activation of ERK1/2 in OC has been shown to enhance tumor progression [49,50]. Activation of the ERK1/2 pathway has also been involved in tumor cell survival by coupling survival stimulus to transcription factors controlling gene expression. For example, higher levels of phospho-ERK1/2 in OVCAR3 cells were associated with increased resistance to cisplatin [51]. In addition, the resistance to paclitaxel can be partially obliterated when ERK1/2 activity is inhibited [52]. The correlation between ERK1/2 activation and Mcl-1 expression in tumor samples from patients with HGSOC suggest that the ERK1/2/Mcl-1 pathway likely exerts a protective anti-apoptotic effect to tumor cells and is biologically relevant.

Our data indicate that the Elk-1 transcription factor is an important regulator of ascites-induced Mcl-1 expression. OC ascites induced a rapid (within 30 min) phosphorylation of Elk-1 in tumor cells. Although other transcription factors such as Stat3 and NF-κB have been reported to regulate Mcl-1 expression [31], it appears that Elk-1 is critical in OC cells as evidenced by the fact that siRNA inhibition of Elk-1 almost completely abolished ascites-induced Mcl-1 upregulation. In accordance with our results, Elk-1-dependent regulation of Mcl-1 expression has been described with other types of cancer [27,28]. Additional studies have shown that Elk-1 is directly phosphorylated by ERK1/2 [28] and therefore support our findings that ascites induce phosphorylation of not only ERK1/2 but also Elk-1.

We have previously shown that soluble factors present in OC ascites engage αvβ5 integrin to induce a FAK-dependent Akt activation that contributes to protect cells from TRAIL-induced apoptosis [13]. Here, we demonstrate that ERK1/2 activation, which contributes to decrease TRAIL-induced apoptosis, is independent from ascites-mediated FAK activation as shown by the fact that the knockdown of FAK does not affect ERK1/2 and Elk-1 phosphorylation. Although growth factor receptors such as EGFR and PDGFR can often activate the ERK pathway [25], and ligands of these receptors are present in OC ascites [53], we do not believe that the ascites-mediated upregulation of Mcl-1 is dependent on these receptors because we previously shown that the inhibition of EGFR and PDGFR does not alter the prosurvival activity of ascites [13].

Our findings suggest that OC ascites activate multiple signaling pathways to inhibit TRAIL-induced apoptosis and each pathway may contribute to a different level to ascites-mediated protection from TRAIL depending, at least in part, on the cell context. Although the significance of these *in vitro* observations in regard to the clinic has yet to be determined, we propose that ascites, by activating different survival pathways in tumor cells, contribute to the persistence of tumor cells during treatment and the occurrence of resistance. This has implication from a therapeutic standpoint. Targeting the tumor environment could be an important strategy to sensitize OC cells to chemotherapy.

## Materials and methods

### Cell culture and reagents

The human OC cell lines CaOV3 and OVCAR3 were obtained from the American Type Culture Collection (Manassas, VA) and maintained in a humidified 5% CO_2_ incubator at 37°C. Cells were passaged twice weekly. OVCAR3 cells were maintained in RPMI-1640 (Wisent, St-Bruno, QC, Canada) supplemented with 20% FBS, insulin (10 mg/L), glutamine (2 mM) and antibiotics. CaOV3 cells were cultured in DMEM/F12 (Wisent) supplemented with 10% FBS, 2 mM glutamine and antibiotics. TRAIL was purchased from PeproTech (Rocky Hill, NJ). Acellular ascites fractions OVC415, OVC508, OVC509, OVC551 were obtained at the time of initial cytoreductive surgery from women with advanced serous ovarian carcinomas. Samples were supplied by the Banque d’échantillons biologiques (seins/ovaires) et de données de Sherbrooke as part of the Banque de tissus et de données du Réseau de Recherche en Cancer des Fonds de Recherche en Santé du Québec (FRSQ) affiliated to the Canadian Tumor Repository Network (CTRNet). HRP-conjugated anti-mouse and -rabbit antibodies, Akt, Bcl-X_L_, Elk-1, phospho-ERK1/2 (Thr202/Tyr204), Mcl-1, FAK, phospho-FAK and phospho-Elk-1 (Ser383) antibodies were purchased from Cell Signaling. Antibodies for phospho-Akt (Ser473) were from Life Technologies (Burlington, ON). Bcl-2 antibody was purchased from Dako (Burlington, ON). ERK antibody was from Santa Cruz Biotech (Santa Cruz, CA). PI3K inhibitor LY294002 and MEK inhibitor U0126 were purchased from EMD (Billerica, MA). Tubulin antibody, actinomycin D and propidium iodide were purchased from Sigma-Aldrich (Oakville, ON). Actinomycin D was dissolved in dimethyl sulfoxide at a concentration of 10 mM and stored at −20°C.

### Quantitative real time PCR

Total RNA was extracted from CaOV3 and OVCAR3 cells using TRIzol reagent (Life Technologies) according to the manufacturer’s protocol and subjected to reverse transcription (RT) with oligodT from Promega (Madison, WI) and MMULV reverse transcriptase enzyme. RNA concentrations were quantified by measurement of absorbance at 260 nm. The integrity of the cDNA was assessed with the Taqman gene expression assays (Life Technologies), done on *RPLPO* housekeeping gene. Each sample was normalized to the housekeeping gene levels. Mcl-1 primers were from Life Technologies (gene expression assay hs03043899, cat # 4331182). Cycle conditions for all PCRs were as follow: an initial incubation of 2 min at 95°C followed by 35 cycles at 94°C 30 s, 55°C 30 s, 72°C 60 s. The amplification occurred for 2 min at 72°C. PCR products quantification was performed as previously described in collaboration with Dr C. Asselin (université de Sherbrooke) [41].

### Apoptosis assays

Analysis of apoptosis was performed by quantification of the sub-G1 peak by flow cytometry as previously described [41]. Propidium iodide staining for DNA fragmentation was done by fixing cells and staining them with propidium iodide for DNA analysis content as previously described [41]. A total of 10,000 events were analyzed by flow cytometry and the percentage of hypodiploid cells was measured using a BD FACScalibur flow cytometer (BD Biosciences, ON, Canada).

### Western blot analysis

Cells were harvested and washed with ice-cold PBS. Whole cell extracts were prepared in lysing buffer (glycerol 10%, Triton X-100 1%, TRIS 10 mM pH 7.4, NaCl 100 mM, EGTA 1 mM, EDTA 1 mM, SDS 0.1%) containing protease inhibitors (0.1 mM AEBSF, 5 μg/ml pepstatin, 0.5 μg/ml leupeptin and 2 μg/ml aprotinin) and phosphatase inhibitors (Na_4_P_2_O_7_ 20 mM, NaF 1 mM, Na_3_VO_4_ 2 mM). Proteins were separated by 12% SDS-PAGE gels. Proteins were transferred to PVDF membranes (Roche, Laval, Québec, Canada) by electroblotting, and immunoblot analysis was performed as previously described [17]. All primary antibodies were incubated overnight at 4°C in 5% fat-free milk. Proteins were visualized by enhanced chemiluminescence (GE Healthcare, Baie d’Urfé, Québec, Canada).

### siRNA transfections

The Fluorescein-labeled Luciferase GL2 duplex or a non-target (scrambled) siRNAs used as a control were from Dharmacon Research (Lafayette, CO). Cells (6 × 10^4^) were seeded in 6-well plates and allowed to adhere for 24 h. Cells (50% confluent) were transfected with a mixture containing Lipofectamine 2000™ (Life Technologies), optiMEM (Life Technologies) and siRNA (10 μM). The siRNAs/Lipofectamine complex was then added to the media of 6-well plates containing cells. Cells were incubated for 4–6 h at 37°C in a CO_2_ incubator and medium containing FBS was then added. The Mcl-1 and FAK siRNAs were from Dharmacon Research, Akt siRNA from Cell Signaling and Elk-1 siRNA from Santa Cruz.

### Immunohistochemistry staining

TMAs were acquired from the Pan-canadian platform for the development of biomarker-driven subtype specific management of ovarian carcinoma (COEUR study). Sections were deparaffinized in citrate buffer containing 0.05% Tween at 97°C for 20 min, washed with PBS and incubated with 3% peroxide. After treatment, slides were submerged in a citrate buffer (0.01 M citric acid, pH 6.0) for 15 min, and incubated with a protein blocking serum-free reagent (Dako Canada). The TMAs were stained by an immunoperoxidase method using an automated tissue immunostainer (Dako Canada) with DABchromogen. The TMAs counter stained with hematoxilin and were visualized by light microscopy at 20× magnification and scored by two blinded independent observers using the H-score method with an inter-rating > 90%. An intensity score of 0–3 was multiplied by the percentage of tumor cells stained to obtain the H-score. *P* values were calculated by the Mann–Whitney test.

### Statistical analysis

Statistical comparisons between two groups were performed using the Mann–Whitney or Student’s *t*-test. The correlation between phosphor-ERK1/2 and Mcl-1 expression in tissue section was determined by the Spearman correlation test. Statistical significance was indicated by *P* < 0.05.

## Competing interests

The authors report no conflict of interest.

## Authors' contributions

NG-K participated in the design of the study and performed most of the experiments. IM was responsible for obtaining the ascites, performing the immunohistochemistry staining with the TMA and obtaining the clinical data. DL performed the long term cell viability assays. CR participated in the design of the study and helped to draft the manuscript. AP conceived the study, participated in its design and drafted the manuscript. All authors read and approved the final manuscript.

## Supplementary Material

Additional file 1 **Figure S1.** - (A) Time course of Mcl-1 protein expression in OVCAR3 cells following addition of 10% OVC509 and OVC551 ascites to the cell culture media. Cells were incubated with ascites for 2 h and 4 h and proteins were extracted. Immunoblots were probed with anti-Mcl-1 and anti-tubulin antibodies (as a loading control). (B) Real-time PCR analysis of Mcl-1 transcript levels from CaOV3 cells incubated in the presence or absence of OVC415 and OVC509 ascites (10%) and actinomycin D. Results were standardized using primers of the housekeeping gene RPLPO. Results are expressed as fold change relative to basal levels observed in cells incubated in the absence of ascites. (C) OVCAR3 cells were treated with protein synthesis inhibitor cycloheximide 2 h before addition of OVC509 ascites. Lysates were obtained after 3 h and immunoblot analysis of Mcl-1 were performed. **Figure S2.** – (A) CaOV3 cells were transfected with vehicle, Mcl-1 or control siRNA. Mcl-1 knockdown was assessed by immunoblot analysis 24 h and 48 h after transfection. ERK1/2 was used as a loading control. (B) CaOV3 were transfected as described and Bcl-2 and Bcl-XL expression were determined 24 h after transfection of Mcl-1 siRNA to ensure that Mcl-1 knockdown does not altered Bcl-2 and Bcl-XL expression. **Figure S3.** – (A) CaOV3 cells were incubated with various ascites (10%) for 2 h. Akt phosphorylation and expression were then determined by immunoblot. (B) CaOV3 cells were incubated with OVC 439 ascites and Akt phosphorylation and expression were assessed at 2 h and 4 h after addition of ascites. Click here for file

## References

[B1] Partridge EE Barnes MN Epithelial ovarian cancer: prevention, diagnosis, and treatment CA Cancer J Clin 1999 49 297 320 10.3322/canjclin.49.5.297 11198956

[B2] Ozols RF Bookman MA Connolly DC Daly MB Godwin AK Schilder RJ Xu X Hamilton TC Focus on epithelial ovarian cancer Canc Cell 2004 5 19 24 10.1016/S1535-6108(04)00002-9 14749123

[B3] NCI SEER Cancer stat fact sheet: cancer of the ovary 2010 National Cancer Institute http://seer.cancer.gov/statfacts/html/ovary.html

[B4] Canistra SA Cancer of the ovary N Engl J Med 2004 351 2519 2529 10.1056/NEJMra041842 15590954

[B5] Bast RC Jr Hennessy B Mills GB The biology of ovarian cancer: new opportunities for translation Nat Rev Cancer 2009 9 415 428 10.1038/nrc2644 19461667 PMC2814299

[B6] Lane D Matte I Rancourt C Piché A The prosurvival activity of ascites against TRAIL is associated with a shorter disease-free interval in patients with ovarian cancer J Ovarian Res 2010 3 1 10 10.1186/1757-2215-3-1 20157422 PMC2821314

[B7] Meads MB Gatenby RA Dalton WS Environment-mediated drug resistance: a major contributor to minimal residual disease Nat Rev Cancer 2009 9 665 674 10.1038/nrc2714 19693095

[B8] Kassis J Klominek J Kohn EC Tumor microenvironment: what can effusions teach us? Diagn Cytopathol 2005 33 316 319 10.1002/dc.20280 16240401

[B9] Schauer IG Sood AK Mok S Liu J Cancer-associated fibroblasts and their putative role in potentiating the initiation and development of epithelial ovarian cancer Neoplasia 2011 13 393 405 21532880 10.1593/neo.101720 PMC3084616

[B10] Whiteside TL The tumor microenvironment and its role in promoting tumor growth Oncogene 2008 27 5904 5912 10.1038/onc.2008.271 18836471 PMC3689267

[B11] Müerköster S Wegehenkel K Arlt A Witt M Sipos B Kruse ML Sebens T Klöppel G Kalthoff H Fölsch UR Schäfer H Tumor stroma interactions induce chemoresistance in pancreatic ductal carcinoma cells involving increased secretion and paracrine effects of nitric oxide and interleukin-1β Cancer Res 2004 64 1331 1337 10.1158/0008-5472.CAN-03-1860 14973050

[B12] Perez LE Parquet N Shain K Nimmanapalli R Alsina M Anasetti C Dalton W Bone marrow stroma confers resistance to Apo2 ligand/TRAIL in multiple myeloma in part by regulating c-FLIP J Immunol 2008 180 1545 1555 18209050 10.4049/jimmunol.180.3.1545

[B13] Lane D Goncharenko-Khaider N Rancourt C Piché A Ovarian cancer ascites protects from TRAIL-induced cell death through αvβ5 integrin-mediated focal adhesion kinase and Akt activation Oncogene 2010 29 3519 3531 10.1038/onc.2010.107 20400979

[B14] Pontiggia O Sampayo R Raffo D Motter A Xu R Bissell MJ Joffé EB Simian M The tumor microenvironment modulates tamoxifen resistance in breast cancer: a role for soluble stromal factors and fibronectin through β1 integrin Breast Cancer Res Treat 2012 133 459 471 10.1007/s10549-011-1766-x 21935603 PMC3719875

[B15] Meunier L Puiffe ML Le Page C Filali-Mouhim A Chevrette M Tonin PN Provencher DM Mes-Masson AM Effect of ovarian cancer ascites on cell migration and gene expression in an epithelial ovarian cancer in vitro model Transl Oncol 2010 3 230 238 20689764 10.1593/tlo.10103 PMC2915414

[B16] Puiffe ML Le Page C Filali-Mouhim A Zietarska M Ouellet V Tonin PN Chevrette M Provencher DM Mes-Masson AM Characterization of ovarian cancer ascites on cell invasion, proliferation, spheroid formation, and gene expression in an in vitro model of epithelial ovarian cancer Neoplasia 2007 9 820 829 10.1593/neo.07472 17971902 PMC2040209

[B17] Lane D Robert V Grondin R Rancourt C Piché A Malignant ascites protect against TRAIL-induced apoptosis by activating the PI3K/Akt pathway in human ovarian carcinoma cells Int J Cancer 2007 121 1227 1237 10.1002/ijc.22840 17534891

[B18] Mills GB May C McGill M Roifman CM Mellors A A putative new growth factor in ascitic fluid from ovarian cancer patients: identification, characterization, and mechanism of action Cancer Res 1988 48 1066 1071 3422589

[B19] Mills GB May C Hill M Campbell S Shaw P Marks A Ascitic fluid from human ovarian cancer patients contains growth factors necessary for intraperitoneal growth of human ovarian adenocarcinoma cells J Clin Invest 1990 86 851 855 10.1172/JCI114784 2394835 PMC296802

[B20] Richardson M Gunawan J Hatton MW Seidlitz E Hirte HW Singh G Malignant ascites fluids (MAF), including ovarian-cancer-associated MAF, contains angiostatin and other factor(s) which inhibit angiogenesis Gynecol Oncol 2002 86 279 287 10.1006/gyno.2002.6760 12217749

[B21] Xu Y Gaudette DC Boynton JD Frankel A Fang XJ Sharma A Hurteau J Casey G Goodbody A Mellors A Holub BJ Mills GB Characterization of an ovarian cancer activating factor in ascites of ovarian cancer patients Clin Cancer Res 1995 1 1223 1232 9815916

[B22] Yamada T Sato K Komachi M Malchinkhuu E Tobo M Kimura T Kuwabara A Yanagita Y Ikeya T Tanahashi Y Ogawa T Ohwada S Morishita Y Ohta H Im DS Tamoto K Tomura H Okajima F Lysophosphatidic acid (LPA) in malignant ascites stimulates motility of human pancreatic cancer cells through LPA1 J Biol Chem 2004 279 6595 6605 14660630 10.1074/jbc.M308133200

[B23] Giuntoli RL Webb TJ Zoso A Rogers O Diaz-Montes TP Bristow RE Oelke M Ovarian cancer-associated ascites demonstrates altered immune environment: implications for antitumor immunity Anticancer Res 2009 29 2875 2884 19661290

[B24] Ahmed N Riley C Oliva K Rice G Quinn M Ascites induces modulation of α6β1 integrin and urokinase plasminogen activator receptor expression and associated functions in ovarian carcinoma Br J Cancer 2005 92 1475 1485 10.1038/sj.bjc.6602495 15798771 PMC2362012

[B25] McCubrey JA Steelman LS Chappell WH Abrams SL Wong EW Chang F Lehmann B Terrian DM Milella M Tafuri A Stivala F Libra M Basecke J Evangelisti C Martelli AM Franklin RA Roles of the RAF/MEK/ERK pathway in cell growth, malignant transformation and drug resistance Biochim Biophys Acta 2007 1773 1263 1284 10.1016/j.bbamcr.2006.10.001 17126425 PMC2696318

[B26] Boucher MJ Morisset J Vachon PH Reed JC Lainé J Rivard N MEK/ERK signaling pathway regulates the expression of Bcl-2, Bcl-XL, and Mcl-1 and promotes survival of human pancreatic cancer cells J Cell Biochem 2000 79 355 369 10.1002/1097-4644(20001201)79:3<355::AID-JCB20>3.0.CO;2-0 10972974

[B27] Balmanno K Cook SJ Tumor cell survival signalling by the ERK1/2 pathway Cell Death Differ 2009 16 368 377 10.1038/cdd.2008.148 18846109

[B28] Booy EP Henson ES Gibson SB Epidermal growth factor regulates Mcl-1 expression through the MAPK-ELK-1 signalling pathway contributing to cell survival in breast cancer Oncogene 2011 30 2367 2378 10.1038/onc.2010.616 21258408 PMC3145838

[B29] Leu CM Chang C Hu C Epidermal growth factor (EGF) suppresses staurosporine-induced apoptosis by inducing mcl-1 via the mitogen-activated protein kinase pathway Oncogene 2000 19 1665 1675 10.1038/sj.onc.1203452 10763823

[B30] Schubert KM Duronio V Distinct roles for extracellular-signal-regulated protein kinase (ERK) mitogen-activated protein kinases and phosphatidylinositol 3-kinase in the regulation of Mcl-1 synthesis Biochem J 2001 356 473 480 10.1042/0264-6021:3560473 11368774 PMC1221858

[B31] Thomas LW Lam C Edwards SW Mcl-1; the molecular regulation of protein function FEBS Lett 2010 584 2981 2989 10.1016/j.febslet.2010.05.061 20540941

[B32] Coloff JL Macintyre AN Nichols AG Liu T Gallo CA Plas DR Rathmell JC Akt-dependent glucose metabolism promotes Mcl-1 synthesis to maintain cell survival and resistance to Bcl-2 inhibition Cancer Res 2011 71 5204 5213 10.1158/0008-5472.CAN-10-4531 21670080 PMC3148426

[B33] Dong L Jiang CC Thorne RF Croft A Yang F Liu H de Bock CE Hersey P Zhang XD Ets-1 mediates upregulation of Mcl-1 downstream of XBP-1 in human melanoma cells upon ER stress Oncogene 2011 30 3716 3726 10.1038/onc.2011.87 21423203 PMC3163261

[B34] Kobayashi S Werneburg NW Bronk SF Kaufmann SH Gores GJ Interleukin-6 contributes to Mcl-1 up-regulation and TRAIL resistance via an Akt-signaling pathway in cholangiocarcinoma cells Gastroenterology 2005 128 2054 2065 10.1053/j.gastro.2005.03.010 15940637

[B35] Kim SH Ricci MS El-Deiry WS Mcl-1: a gateway to TRAIL sensitization Cancer Res 2008 68 2062 2064 10.1158/0008-5472.CAN-07-6278 18381408

[B36] Clohessy JG Zhuang J de Boer J Gil-Gómez G Brady HJ Mcl-1 interacts with truncated Bid and inhibits its induction of cytochrome c release and its role in receptor-mediated apoptosis J Biol Chem 2006 281 5750 5759 16380381 10.1074/jbc.M505688200

[B37] Han J Goldstein LA Gastman BR Rabinowich H Interrelated roles for Mcl-1 and BIM in regulation of TRAIL-mediated mitochondrial apoptosis J Biol Chem 2006 281 10153 10163 10.1074/jbc.M510349200 16478725

[B38] Ashkenazi A Directing cancer cells to self-destruct with pro-apoptotic receptor agonists Nat Rev Drug Discov 2008 7 1001 1012 10.1038/nrd2637 18989337

[B39] Goncharenko-Khaider N Lane D Matte I Rancourt C Piché A Targeted ovarian cancer treatment: the TRAILs of resistance Am J Cancer Res 2012 2 75 92 22206047 PMC3236573

[B40] Youle RJ Strasser A The BCL-2 protein family: opposing activities that mediate cell death Nat Rev Mol Cell Biol 2008 9 47 59 10.1038/nrm2308 18097445

[B41] Goncharenko-Khaider N Lane D Matte I Rancourt C Piché A The inhibition of Bid expression by Akt leads to resistance to TRAIL-induced apoptosis in ovarian cancer cells Oncogene 2010 29 5523 5536 10.1038/onc.2010.288 20661217 PMC3007125

[B42] Dodier P Piché A Bcl-XL is functionally non-equivalent for the regulation of growth and survival in human ovarian cancer cells Gynecol Oncol 2006 100 254 263 10.1016/j.ygyno.2005.08.028 16188300

[B43] Lane D Cartier A L’Espérance S Côté M Rancourt C Piché A Differential induction of apoptosis by tumor necrosis factor-related apoptosis-inducing ligand (TRAIL) in human ovarian carcinoma cells Gynecol Oncol 2004 93 594 604 10.1016/j.ygyno.2004.03.029 15196850

[B44] Favata MF Horiuchi KY Manos EJ Daulerio AJ Stradley DA Feeser WS Van Dyk DE Pitts WJ Earl RA Hobbs F Copeland RA Magolda RL Scherle PA Trzaskos JM Identification of a novel inhibitor of mitogen-activated protein kinase J Biol Chem 1998 27 18623 18632 9660836 10.1074/jbc.273.29.18623

[B45] Brotin E Meryet-Figuière M Simonin K Duval RE Villedieu M Leroy-Dudal J Saison-Behmoaras E Gauduchon P Denoyelle C Poulain L Bcl-XL and MCL-1 constitute pertinent targets in ovarian carcinoma and their concomitant inhibition is sufficient to induce apoptosis Int J Cancer 2010 126 885 895 19634140 10.1002/ijc.24787

[B46] Shigemasa K Katoh O Shiroyama Y Mihara S Mukai K Nagai N Ohama K Increased MCL-1 expression is associated with poor prognosis in ovarian carcinomas Jpn J Cancer Res 2002 93 542 550 10.1111/j.1349-7006.2002.tb01289.x 12036450 PMC5927039

[B47] Baekelandt M Holm R Nesland JM Tropé CG Kristensen GB Expression of apoptosis-related proteins is an independent determinant of patient prognosis in advanced ovarian cancer J Clin Oncol 2000 18 3775 3781 11078490 10.1200/JCO.2000.18.22.3775

[B48] Simonin K Brotin E Dufort S Dutoit S Goux D N’diaye M Denoyelle C Gauduchon P Poulain L Mcl-1 is an important determinant of the apoptotic response to the BH3-mimetic molecule HA14-1 in cisplatin-resistant ovarian carcinoma cells Mol Cancer Ther 2009 8 3162 3170 10.1158/1535-7163.MCT-09-0493 19887550

[B49] Fujisawa T Joshi BH Puri RK IL-13 regulates cancer invasion and metastasis through IL-13Rα2 via ERK/AP-1 pathway in mouse model of human ovarian cancer Int J Cancer 2012 131 344 356 10.1002/ijc.26366 21858811

[B50] Tanaka Y Kobayashi H Suzuki M Kanayama N Terao T Transforming growth factor-beta1-dependent urokinase up-regulation and promotion of invasion are involved in Src-MAPK-dependent signaling in human ovarian cancer cells J Biol Chem 2004 279 8567 8576 10.1074/jbc.M309131200 14676209

[B51] Lee S Yoon S Kim DH A high nuclear basal level of ERK2 phosphorylation contributes to the resistance of cisplatin-resistant human ovarian cancer cells Gynecol Oncol 2007 104 338 344 10.1016/j.ygyno.2006.08.040 17023032

[B52] Pan ZZ Bruening W Giasson BI Lee VM Godwin AK Gamma-synuclein promotes cancer cell survival and inhibits stress- and chemotherapy drug-induced apoptosis by modulating MAPK pathways J Biol Chem 2002 277 35050 35060 10.1074/jbc.M201650200 12121974

[B53] Matte I Lane D Laplante C Rancourt C Piché A Profiling of cytokines in human epithelial ovarian cancer ascites Am J Cancer Res 2012 2 566 580 22957308 PMC3433103

