# Supplementary material for: Ovarian cancer ascites increase Mcl-1 expression in tumor cells through ERK1/2-Elk-1 signaling to attenuate TRAIL-induced apoptosis
Source: Mol Cancer. 2012 Nov 17;11:84. doi: 10.1186/1476-4598-11-84 (PMC3526430; doi:10.1186/1476-4598-11-84)
Supplement: Additional file 1 — Figure S1. - (A) Time course of Mcl-1 protein expression in OVCAR3 cells following addition of 10% OVC509 and OVC551 ascites to the cell culture media. Cells were incubated with ascites for 2 h and 4 h and proteins were extracted. Immunoblots were probed with anti-Mcl-1 and anti-tubulin antibodies (as a loading control). (B) Real-time PCR analysis of Mcl-1 transcript levels from CaOV3 cells incubated in the presence or absence of OVC415 and OVC509 ascites (10%) and actinomycin D. Results were standardized using primers of the housekeeping gene RPLPO. Results are expressed as fold change relative to basal levels observed in cells incubated in the absence of ascites. (C) OVCAR3 cells were treated with protein synthesis inhibitor cycloheximide 2 h before addition of OVC509 ascites. Lysates were obtained after 3 h and immunoblot analysis of Mcl-1 were performed. Figure S2. – (A) CaOV3 cells were transfected with vehicle, Mcl-1 or control siRNA. Mcl-1 knockdown was assessed by immunoblot analysis 24 h and 48 h after transfection. ERK1/2 was used as a loading control. (B) CaOV3 were transfected as described and Bcl-2 and Bcl-XL expression were determined 24 h after transfection of Mcl-1 siRNA to ensure that Mcl-1 knockdown does not altered Bcl-2 and Bcl-XL expression. Figure S3. – (A) CaOV3 cells were incubated with various ascites (10%) for 2 h. Akt phosphorylation and expression were then determined by immunoblot. (B) CaOV3 cells were incubated with OVC 439 ascites and Akt phosphorylation and expression were assessed at 2 h and 4 h after addition of ascites. [file 1476-4598-11-84-S1.pptx]

## Slide 1
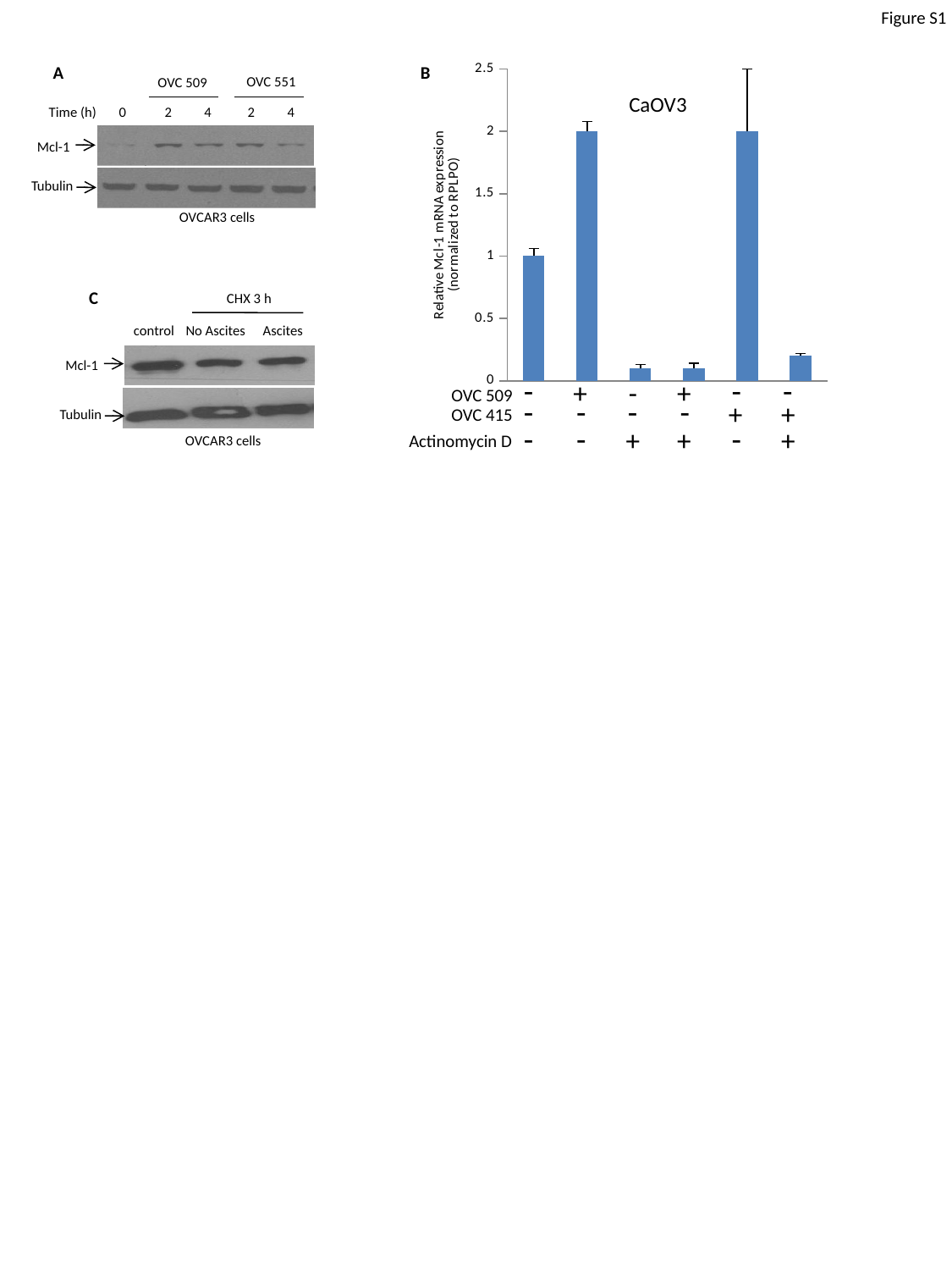

Figure S1
A
### Chart
| Category | |
|---|---|
| control | 1.0 |
| OVC509 | 2.0 |
| Act | 0.1 |
| OVC509+Act | 0.1 |
| OVC415 | 2.0 |
| OVC415+Act | 0.2 |B
OVC 551
OVC 509
CaOV3
Time (h)
0
2
4
2
4
Mcl-1
Tubulin
OVCAR3 cells
C
CHX 3 h
control
No Ascites
Ascites
Mcl-1
-
-
-
+
-
+
OVC 509
-
-
-
-
+
+
OVC 415
Tubulin
-
-
-
+
+
+
Actinomycin D
OVCAR3 cells

## Slide 2
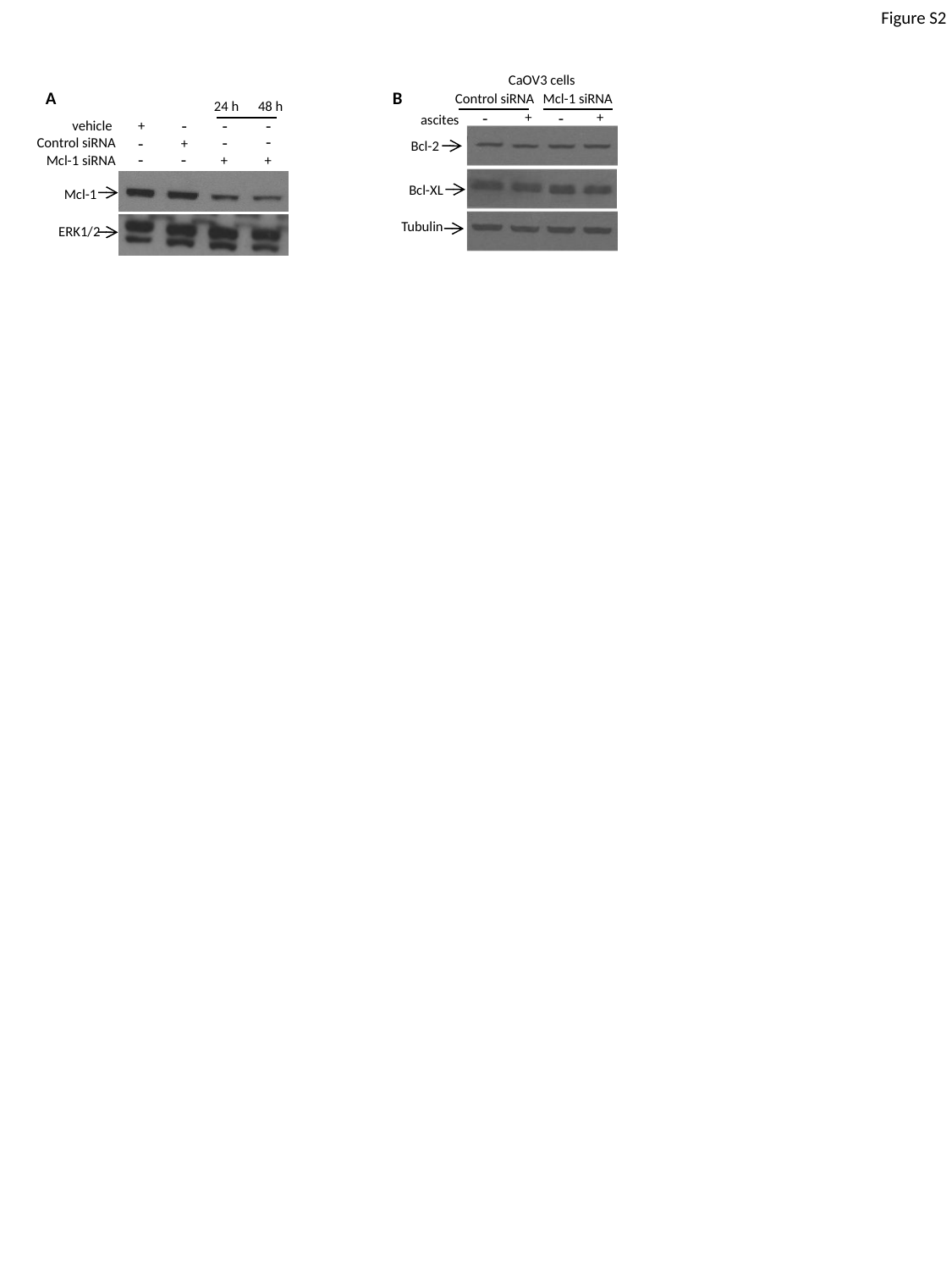

Figure S2
CaOV3 cells
A
B
Control siRNA
Mcl-1 siRNA
24 h
48 h
-
-
+
+
 ascites
-
-
-
vehicle
+
-
-
-
Control siRNA
+
Bcl-2
-
-
+
Mcl-1 siRNA
+
Bcl-XL
Mcl-1
Tubulin
ERK1/2

## Slide 3
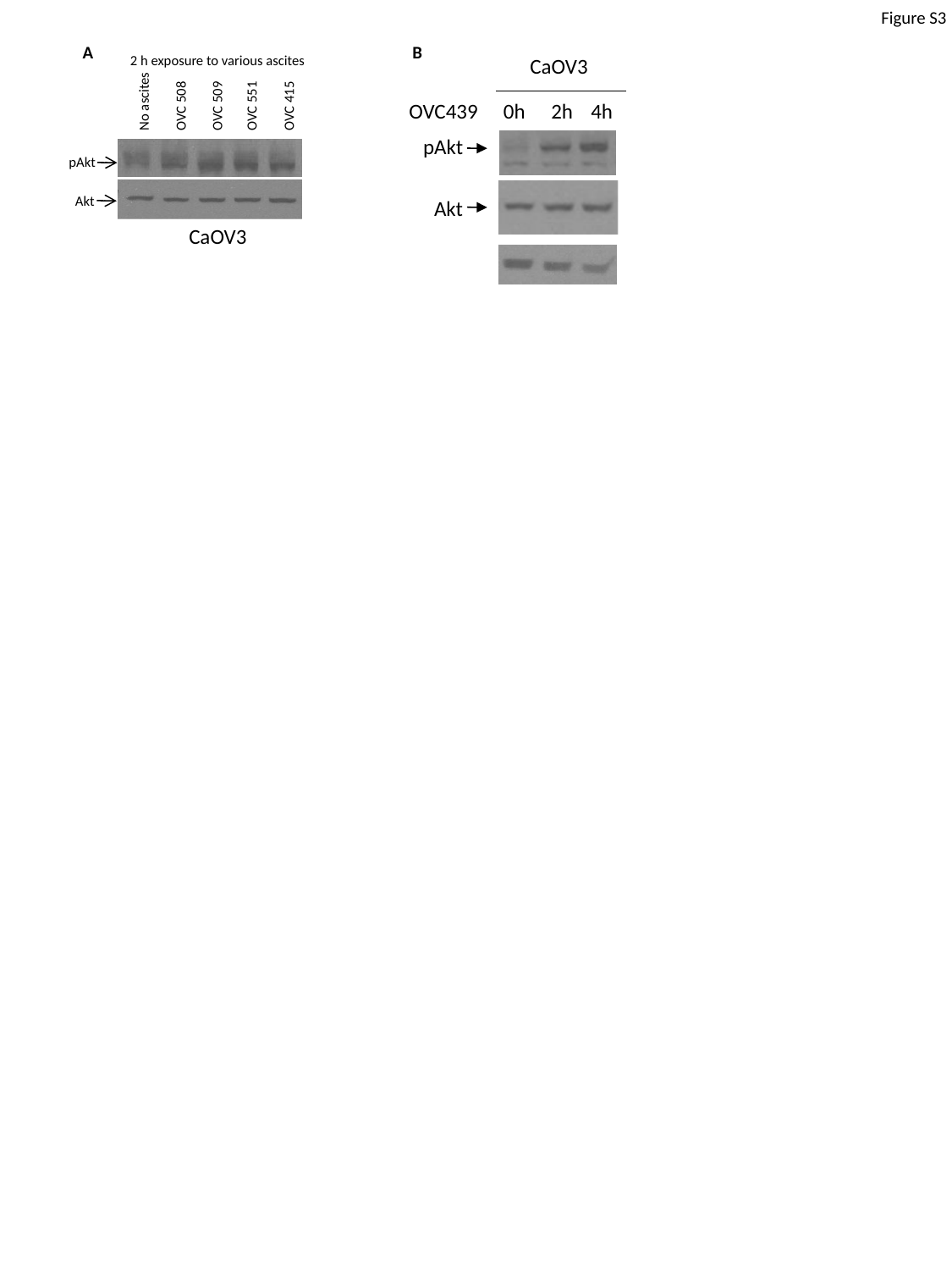

Figure S3
A
B
2 h exposure to various ascites
CaOV3
No ascites
OVC 551
OVC 509
OVC 415
OVC 508
OVC439
0h
2h
4h
pAkt
pAkt
Akt
Akt
CaOV3
